# Comparing safety, performance and user perceptions of a patient-specific indication-based prescribing tool with current practice: a mixed methods randomised user testing study

**DOI:** 10.1136/bmjqs-2024-017733

**Published:** 2024-11-21

**Authors:** Calandra Feather, Jonathan Clarke, Nicholas Appelbaum, Ara Darzi, Bryony Dean Franklin

**Affiliations:** 1 Department of Surgery and Cancer, Imperial College London, London, UK; 2 Dosium Holdings Limited, London, UK; 3 Department of Mathematics, Imperial College London, London, UK; 4 Director, Centre for Medication Safety and Service Quality, Imperial College Healthcare NHS Trust, London, UK; 5 Department of Practice and Policy, UCL School of Pharmacy, London, UK

**Keywords:** Medication safety, Decision support, clinical, Patient safety, Simulation, Randomised controlled trial

## Abstract

**Background:**

Medication errors are the leading cause of preventable harm in healthcare. Despite proliferation of medication-related clinical decision support systems (CDSS), current systems have limitations. We therefore developed an indication-based prescribing tool. This performs dose calculations using an underlying formulary and provides patient-specific dosing recommendations. Objectives were to compare the incidence and types of erroneous medication orders, time to prescribe (TTP) and perceived workload using the NASA Task Load Index (TLX), in simulated prescribing tasks with and without this intervention. We also sought to identify the workflow steps most vulnerable to error and to gain participant feedback.

**Methods:**

A simulated, randomised, cross-over exploratory study was conducted at a London NHS Trust. Participants completed five simulated prescribing tasks with, and five without, the intervention. Data collection methods comprised direct observation of prescribing tasks, self-reported task load and semistructured interviews. A concurrent triangulation design combined quantitative and qualitative data.

**Results:**

24 participants completed a total of 240 medication orders. The intervention was associated with fewer prescribing errors (6.6% of 120 orders) compared with standard practice (28.3% of 120 orders; odds ratio 0.18, p<0.01), a shorter TTP and lower overall NASA-TLX scores (p<0.01). Control arm workflow vulnerabilities included failures in identifying correct doses, applying maximum dose limits and calculating patient-specific dosages. Intervention arm errors primarily stemmed from misidentifying patient-specific information from the medication scenario. Thematic analysis of participant interviews identified six themes: navigating trust and familiarity, addressing challenges and suggestions for improvement, integration of local guidelines and existing CDSS, intervention endorsement, ‘search by indication’ and targeting specific patient and staff groups.

**Conclusion:**

The intervention represents a promising advancement in medication safety, with implications for enhancing patient safety and efficiency. Further real-world evaluation and development of the system to meet the needs of more diverse patient groups, users and healthcare settings is now required.

**Trial registration number:**

NCT05493072.

**WHAT IS ALREADY KNOWN ON THIS TOPIC**

- Indication-based prescribing has the potential to improve prescribing efficiency and patient safety.

**WHAT THIS STUDY ADDS**

- An indication-based, patient-specific prescribing tool used in a simulation setting reduced the incidence of prescribing errors and the time to prescribe compared with standard practice.
- This study provides cumulative validity to the potential benefits of indication-based prescribing tools.

**HOW THIS STUDY MIGHT AFFECT RESEARCH, PRACTICE OR POLICY**

- Future evaluation of such tools in the real-world clinical setting is now required to identify the impact of such tools on clinical outcomes and prescribing workflow.

## Introduction

 Medication errors are the leading cause of preventable harm in healthcare settings worldwide.^1^ An estimated 237 million medication errors occur in England alone every year, with 66 million considered clinically significant.^2^ Avoidable adverse drug events related to these errors are estimated to cost the NHS in excess of £98.5 million per year, consuming 181 626 bed-days and causing 712 deaths.^2^

Medication-related clinical decision support systems (CDSS), often integrated with electronic prescribing (eP), have proliferated over the last few decades. Functionality provided by these systems has typically been limited to alerts pertaining to drug–drug interactions, allergies, duplications and basic dose range checking. A recent systematic review found such systems to be relatively immature, with little to no human factors input during development and a functionality that is largely generic to all patients.^3^ There is therefore an opportunity to improve CDSS for patient safety by integrating patient-specific and medication-specific factors (such as indication for use) and applying human factors and usability engineering to ensure systems are both user-friendly and safe.^4 5^

Indication-based prescribing allows clinicians to select medications and doses that are explicitly linked to the condition being treated, with the aim of improving safety and reducing errors by aligning medication orders with the clinical indication.^6^,^9^ A US study of an indication-based prescribing tool demonstrated improvements in efficiency, medication error rates and user satisfaction.^10^ However, evaluation of similar interventions in different contexts, with different systems and users, is required. Our objectives were to compare the incidence and types of erroneous medication orders, time to prescribe (TTP) and perceived workload, in simulated prescribing tasks with and without the use of a patient-specific, indication-based prescribing intervention. In addition, we sought to identify workflow steps most vulnerable to error and to gain participant feedback regarding use of the intervention.

## Methods

### Study design and setting

We used a simulated, randomised, cross-over exploratory study to compare prescribing with and without use of a prototype CDSS at a large London (UK) teaching NHS Trust, from December 2022 to April 2023. Both quantitative and qualitative methods and analysis were used in a concurrent triangulation design, whereby a combination of methods and outcomes can provide an expanded understanding of the studied phenomena^11 12^ (online supplemental appendix 1). The Trust comprises three hospitals, and serves a wide range of paediatric and adult specialities and uses Cerner^13^ as its primary electronic heath record (EHR) and eP system.

### The intervention

Touchdose is a UK conformity-assessed medical device that integrates with Cerner and other EHR systems to provide users with patient-specific, indication-based dosing recommendations. Touchdose uses medication and indication inputs from prescribers, combined with patient data automatically retrieved from the EHR to perform dosing calculations as needed, to apply clinical logic to an underlying formulary, primarily the British National Formulary (BNF)^14^ and BNF for Children (BNFc).^15^ See online supplemental appendices 2 and 3 for screenshots of the user interface.

### Identification of participants

Participants were a convenience sample of clinicians who were approached if they regularly prescribed medications for hospital inpatients across one or more of the three hospital sites. Targeted sampling was used to recruit both medical and non-medical prescribers, from a wide range of specialities and levels of seniority. We had a target sample of 30; however, recruitment was stopped after 4 months due to lack of clinician availability to participate and time constraints.

### Study procedure

Recruited clinicians were block randomised^16^ by the primary researcher into one of four groups (online supplemental appendix 4) using an online random team generator. Group allocation determined the order in which each participant would complete two sets of five prescribing scenarios (set 1 and set 2) and the order of study arms (control or intervention). Prescribing scenarios (online supplemental appendix 5) were created by a multidisciplinary team of clinicians to test a range of common prescribing skills for both adult and paediatric inpatients. These scenarios included requirements such as ideal body weight calculation, body surface area calculation, route-specific dosing and a maximum dose limit. Less commonly prescribed medications were used to reduce the use of clinicians’ memory to encourage to use clinical resources and calculate doses where necessary.

Prescribing sessions were recorded using a high-definition camera coupled with desktop screen recording to aid collection of timing and workflow data that would not otherwise be feasible to collect in real time. All participants viewed a 4 min introductory training video of the intervention, followed by completing two practice scenarios. These were completed with assistance of the researcher if the participant asked for guidance.

Prescribing scenarios were presented to the participants on paper along with relevant patient and clinical information, for example, patient sex, age, weight, indication for use and relevant medical history. Participants were asked to prescribe one medication per scenario, five using the intervention and five using the usual resources available at the hospital. After a dose was determined, participants manually entered the medication order for the test patient on Cerner.^13^

Following completion of each study arm, participants were asked to score their perceived workload for completing the prescribing scenarios using the NASA Task Load Index (TLX) survey.^17^ This comprises six subjective workload scales: mental demand, physical demand, temporal demand, performance, effort and frustration. On completion of both study arms, they were invited to take part in a brief (10–20 min) semistructured interview. This included questions about their experience using the intervention, potential future features and how they perceived it could be integrated into practice (topic guide in online supplemental appendix 6). Prescribing sessions and subsequent interviews were conducted in an office at the hospital.

### Outcome definitions

Definitions for each outcome measure, adapted from previous work,^18^ are summarised below.

#### Erroneous medication orders and prescribing errors

An ‘erroneous medication order’ was defined as a medication order associated with one or more prescribing errors. A prescribing error was anything that deviated from recommendations in BNF, BNFc and/or local guidelines. Prescribing errors could comprise one or more of the following: wrong dosing (deviation>10% outside recommended dosing range), route, frequency, patient, formulation or brand (where the brand is relevant). We defined large magnitude dosing errors as deviation>25% outside recommended dosing range.

#### Time to prescribe (TTP)

For the first scenario, this was calculated from the time the participant began to read the scenario to the time of task completion, and for subsequent scenarios, from the time they completed the previous scenario and moved on to the next. The end point was when the participant submitted the medication order on the eP system.

#### Prescribing workflows

Workflow steps (such as individual tasks or actions) for both the control and intervention arms were created using previously known or anticipated prescribing workflows and adapted if new unanticipated steps were identified during the study observations. These workflows were then used for hierarchical task analysis as described in the data analysis section.

### Data collection

Participant demographic information was collected before commencement of the session. The researcher kept field notes to assist with analysis of observations and interviews. NASA-TLX questionnaires were completed by the participant after each of the study arms. Retrospective review of the audio-visual recordings were used to collect the timings, workflow and interview data. All quantitative data was entered into an Excel spreadsheet prior to analysis.

Recruitment, consent, randomisation and data collection were conducted by the first author, a female paediatric nurse/researcher and PhD student. She was known to some participants as she was employed in the study hospitals.

### Data analysis

#### Error identification

All potential errors identified by the primary researcher were presented to four pharmacists involved with medication safety research who were blinded to the participant and study arm in which the potential error was observed (see online supplemental appendix 7 for an example). Each potential error was discussed to determine whether it was in fact erroneous and if so, what error type(s) were present, until consensus achieved.

#### Quantitative analysis

Initial descriptive analyses were conducted on all quantitative outcomes. Univariate logistic regression was used to examine association between the variable of interest (study arm) and the incidence of erroneous medication orders, while quantile regression was used to explore the effects across different points in the distribution of ‘TTP’ (eg, lower, median and upper quartiles).^19^ Multivariate logistic and quantile regressions were subsequently conducted as sensitivity analyses to correct for potential residual confounding, controlling for study period and medication set as covariates. Finally, for NASA-TLX scores, we calculated mean scores for overall workload and each individual domain per arm, followed by a Mann-Whitney U test to determine if there was a statistically significant difference between control and intervention arms.^20^ All statistical analysis was conducted using STATA V.18.^21^

#### Qualitative analysis

Audio-visual recordings of semistructured interviews were transcribed verbatim, and reflexive thematic analysis performed on the interview transcripts by the primary researcher, guided by Braun and Clarke’s method.^22^,^24^ This included initial familiarisation of the data by repeated reading of the transcripts, followed by initial code generation. The codes were grouped into themes and the themes then reviewed and refined, with clear definitions and names assigned.

#### Hierarchical task analysis

Hierarchical task analysis was performed by reviewing audio-visual recordings of all erroneous medication orders. This approach allowed the breakdown of the prescribing process into smaller, structured steps, facilitating identification of potential risks or inefficiencies within the workflow and enabling the researcher to trace the likely origin of each error.

#### Triangulation of outcomes and results

The individual outcomes, methods and analysis were considered collectively by the primary researcher. For example, errors are presented in this paper to illustrate not just their incidence but also where in the prescribing workflow they occurred/originated. In addition, the results were synthesised collectively to create and prioritise recommendations for practice and further research, as well as to inform ongoing development and implementation plans for the intervention. Issues identified that had both quantitative and qualitative evidence to support them were prioritised.

### Study registration and reporting

The study is registered on clinicaltrials.gov (reference NCT05493072). It is reported using Consolidated Standards of Reporting Trials and the simulation study extension.^25^

## Results

Data were collected during 24 participant sessions, each comprising two sets of five simulated medication orders, one based on current practice and one using the intervention. Participants comprised 20 doctors and four pharmacist prescribers (table 1).

**Table 1:** Participant demographics

Age	25–34	10	41.70%
	35–44	9	37.50%
	45–54	3	12.50%
	Not stated	2	8.30%
Gender	Female	15	62.50%
	Male	8	33.30%
	Not stated	1	4.20%
Profession	Doctor	20	83.30%
	Pharmacist	4	16.70%
Specialty (self-reported)	Paediatric Emergency	1	4.20%
	PICU	4	16.70%
	Paediatrics	9	37.50%
	Adult	10	41.70%
Participant Grade (self-reported)	Foundation Year 1	1	4.20%
	Senior House Officer	1	4.20%
	ST/CT Years 1–5	5	20.80%
	ST Years 6–8	6	25.00%
	Registrar	1	4.20%
	Clinical Fellow	3	12.50%
	Trust grade	2	8.30%
	Consultant	1	4.20%
	Pharmacist Pay Band 8A	1	4.20%
	Pharmacist Pay Band 8B	3	12.50%
Years using Cerner	< 1 year	6	25.00%
	1–2 years	2	8.30%
	2–3 years	6	25.00%
	3–4 years	2	8.30%
	4–5 years	2	8.30%
	5+years	6	25.00%

Specialty training (ST)/core training (CT) indicate different stages of ST or CT in various medical specialities in the United Kingdom. The number following ‘ST’ or ‘CT’ denotes the specific year of training in the specialty or core training programme.^33^

Adult, adult medical or surgical; PICU, paediatric intensive care unit.

### Prescribing errors

#### Erroneous medication orders

We observed 34 erroneous medication orders (with one or more errors) out of 120 medication orders (28.3%) in the control arm, and eight (6.6%) out of 120 medication orders in the intervention arm. Univariate logistic regression showed the intervention group had significantly lower odds of an erroneous order compared with the control group (OR 0.18; 95% CI 0.08 to 0.41; p<0.01) Sensitivity analyses accounting for both the period and medication set in a multivariate logistic regression model confirmed that the intervention was associated with a statistically significantly lower odds of error (OR 0.16, p<0.01). Overall counts of erroneous medication orders by arm, period and medication set are presented alongside the regression outputs in table 2.

**Table 2:** Univariate and multivariate regression outputs for models examining the relationship between use of the intervention (arm), the covariates (period and set) and the incidence of erroneous medication orders

		Univariate model			Multivariate model		
Erroneous medication orders	n (%)*	OR	95% CI	P value	OR	95% CI	P value
Arm							
**Control**	34 (28.3%)	1	–	–	1	–	–
Intervention	8 (6.6%)	0.18	0.08 to 0.41	<0.01	0.16	0.06 to 0.43	<0.01
Period							
Period 1	12 (10%)	0.33	0.16 to 0.69	<0.01	1.1	0.41 to 3.18	0.79
**Period 2**	30 (25%)	1			1		
Set							
**Set 1**	27 (22.5%)	1	–	–	1	–	–
Set 2	15 (12.5%)	0.49	0.25 to 0.98	0.04	0.43	0.17 to 1.04	0.06

Bold indicates reference category.

* n=number of erroneous medication orders and percentage out of the total 120 medication orders observed per arm.

#### Prescribing errors by type

In the control arm, route errors were observed in 3.3% of all orders, while no wrong patient errors were observed, as shown in table 3. Frequency errors were observed in 2.5% of medication orders and formulation errors in 5.0%. Brand errors were relatively common, accounting for 33.3% of the 12 cases for which the brand name was required and large magnitude errors in 18.3% of cases.

**Table 3:** Number of prescribing errors—by error type

Error	Control (n (%))*	Intervention (n (%))*		
		Intervention Total	Intervention—external to intervention component of workflow	Intervention—within intervention component of workflow
Dose error	31 (25.8%)	8 (6.6%)	7 (5.8%)	1 (0.8%)
Large magnitude dose errors	22 (18.3%)	6 (5.0%)	6 (5.0%)	0 (0%)
Route error	4 (3.3%)	3 (2.5%)	3 (2.5%)	0 (0%)
Patient error	0 (0%)	1 (0.8%)	1 (0.8%)	0 (0%)
Frequency error	3 (2.5%)	0 (0%)	0 (0%)	0 (0%)
Formulation error	6 (5.0%)	3 (2.5%)	3 (2.5%)	0 (0%)
Brand error	4 (33.3%)	0 (0%)	0 (0%)	0 (0%)

* n=number of prescribing errors and percentage of the total 120 medication orders observed per arm, with the exception of brand errors that were relevant to 12 orders per arm where brand specificity was required.

In contrast, in the intervention arm, route errors occurred in 2.5%, and a single patient error was noted (0.8% of orders). Frequency and brand errors were entirely absent, while formulation errors occurred in 2.5%. Large magnitude errors occurred in only 5%.

### Time to prescribe (TTP)

Two participants, with a total of 20 medication orders, were excluded from TTP analysis due to video recording failure. Therefore, a total of 220 medication orders were included in analysis.

The median TTP for a medication order in the control arm was 198 s (IQR 148–280), compared with 164 s (IQR 131–222) in the intervention arm. Univariate quantile regression analysis showed no significant difference in the lower quartile TTP between the control and intervention arms (−17 s, CI −35.1 to 1.1; p=0.07). There was, however, a statistically significant decrease in median and upper quartile TTP in the intervention arm (median: −35 s, CI −62.4 to −7.5; p=0.01 and upper quartile: −56 s, CI −103.8 to −8.3; p=0.02). This statistically significant difference remained following secondary sensitivity analysis using multivariate quantile regression analysis. Full regression outputs and a box plot are available in online supplemental appendices 8–11.

### Hierarchical task analysis

**Figure 1:**
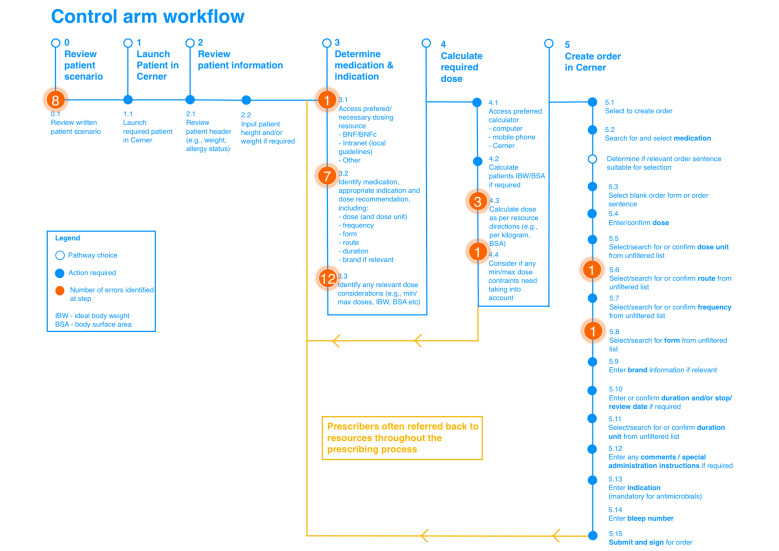
Control arm workflow and hierarchical task analysis, identifying erroneous steps observed. BNF, British National Formulary; BNFc, BNF for Children.

In the control arm, various steps in the prescribing workflow were identified as causes of error, including failure to identify correct doses, apply maximum dose limits and calculate appropriate dosages based on patient-specific factors. A significant proportion of errors were attributed to step 3 ‘determine medication and indication’, the step that required prescribers to access and identify the appropriate medication, relevant indication and dose recommendation for the patient (figure 1 and online supplemental appendix 12).

**Figure 2:**
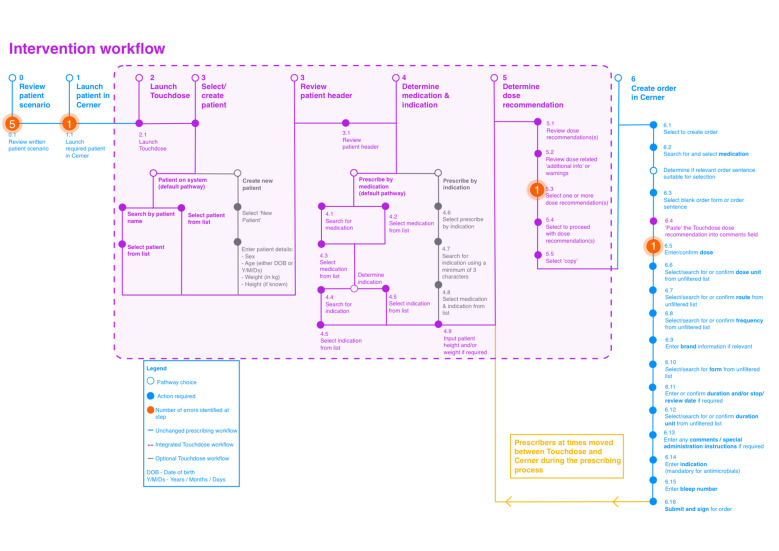
Intervention workflow and hierarchical task analysis, identifying erroneous steps observed.

In contrast, errors in the intervention arm primarily derived from a failure to identify patient-specific information from the medication scenario (five of eight errors). The remaining three errors were a failure to launch the correct patient in Cerner, failure to input a single dose rather than a range and selection of an incorrect dose for the specified route (figure 2 and online supplemental appendix 13).

### Participant feedback

#### NASA TLX scores

NASA-TLX scores revealed that 23 of 24 users perceived a lower task load in the intervention arm compared with control. Mann-Witney U tests demonstrated statistically significant differences between the intervention and control arms in overall task load (41.5 vs 57.2; p<0.01), mental demand (9.5 vs 12.9; p=0.03), effort (8.3 vs 12.1; p<0.01) and frustration (6.2 vs 10.7; p<0.01). There was no statistically significant difference in physical demand, temporal demand and perceived performance. The mean NASA-TLX scores and box and whisker plots for each domain by study arm are presented in online supplemental appendices 14–16.

#### Participant insights

We identified six themes reflecting a variety of participant insights following use of the intervention. These mostly concerned practical considerations that can be used for the continued development of the intervention prior to implementation, prioritisation of future features and proposed patient groups that might most benefit.

##### Navigating trust and familiarity: interplay between existing and new systems

Participants expressed mixed experiences with existing systems, citing positive aspects such as locally created care sets and negative experiences due to complexity. Comparisons were drawn between the intervention and other systems; participants noted similarities in functionality but also highlighted that these systems had limitations.

you are not sure [referring to other eP systems] if it’s done it on the right weight or if it’s calculated the body surface or whatever. Participant 15

What I would say which is really worrying is that these weights are often massively wrong [referring to the weight on the patient header in Cerner]. Participant 19

have we updated the weight because it could be completely different to what the computer is calculating on and things like that coz that’s what I’ve found sometimes with [system used in intensive care unit at study site]. Participant 5

Concerns about trust and reliability of a new system were apparent when articulating known pitfalls of existing prescribing tools/systems. There was therefore a nuanced relationship among trust, familiarity and reliance on different prescribing resources. Participants expressed a range of sentiments regarding their trust in the intervention versus existing resources such as Cerner and the BNF. Some participants expressed a preference for familiar systems, indicating a need for time to adapt and build trust in new technologies. Others expressed a desire to cross-reference the intervention’s recommendations with the BNF, highlighting a need for reassurance and validation of information. Additionally, questions arose about responsibility, with participants questioning whether errors, were they to be encountered in clinical use, would be attributable to the prescriber or to the intervention.

I think that the biggest thing was trusting that the information was being pulled over correctly. Participant 8

Sorry I’m just more used to Cerner. Participant 12

##### Addressing challenges and suggestions for improvement in intervention integration

Participants highlighted various negative experiences and made new feature suggestions that would improve use of the intervention, particularly in comparison to existing systems such as Cerner and the BNF. Participants expressed concerns about the reliability of Cerner’s weight inputs and the potential for errors when transcribing information from the intervention interface to Cerner’s medication order form.

because you have to transfer the information manually I think I feel this is quite like error prone. Participant 7

Additionally, there were suggestions for improvements, such as providing renal and hepatic function warnings and adjustments, allergies and pregnancy status. Concerns also arose about technical issues such as wireless internet connectivity, and usability issues such as small text size and unclear navigation.

##### Integration of local guidelines and existing CDSS

Participants enquired about how the intervention will integrate with existing care sets, indicating an assumption of seamless compatibility with established Cerner workflows. Discussions also revolved around the utility of order sentences and power plans, especially for specific drugs such as vitamin D, highlighting the potential for the intervention to generate tailored recommendations based on patient-specific inputs. Additionally, participants expressed a preference for links to local guidelines over the BNF for antimicrobial prescribing, emphasising the importance of aligning with institutional practices.

I think I’d still have to check this [local guideline] unless when I typed in conjunctivitis it popped up with like a first-choice therapy box and a secondary therapy box. Participant 1

When participants were informed of the intervention developer’s intention to integrate the local guidelines for certain medications, this was received favourably:

that would definitely be super helpful. Participant 17

especially if you have a link even for like more cautious one [prescriber] I can see the trust guideline it I guess like save me from having to like find the right guideline which itself can be quite tricky. Participant 20

##### Intervention endorsement: enhancing safety, efficiency and user experience

Participants overwhelmingly highlighted a positive experience and provided endorsement of the intervention system regarding various aspects, particularly in terms of safety, efficiency and ease of use. Participants appreciated the system’s potential to reduce prescribing errors, streamline workflows by providing all necessary information in one place and remove the need for complex calculations such as for body surface area. They also praised the user-friendly interface and layout, noting clarity, ease of navigation and better presentation of BNF data compared with current methods.

##### Search by indication

Participants generally approved of the concept of ‘search by indication’, to enable an indication-first prescribing workflow, a future feature of the intervention that was discussed during the semistructured interview. This feature was present in the intervention but no participants selected this function during the study. Most recognised its potential utility, especially for exploring alternative treatment options and navigating through complex medication choices. Participants highlighted scenarios where searching by indication first (rather than medication), for medication groups such as antimicrobials or antiemetics, could further enhance clinical decision-making and improve patient care. However, there were caveats and uncertainties expressed by some participants. Concerns were raised about the need for alignment with local guidelines, the preference for searching by medication rather than indication and the reliance on other resources such as the app used locally for antimicrobial prescribing guidelines.

##### Targeting specific patient and staff groups

Many patient groups that might most benefit from the intervention were suggested; these included paediatrics, the elderly and adults post heart attack or kidney transplant, those with renal or hepatic failure, breastfeeding or with obesity. The most frequently mentioned was paediatrics (eight participants), mainly due to the high frequency of patient-specific dose calculations. Staff groups that might benefit included all doctors with many specifying junior doctors in particular. Nurse prescribers, intensive care doctors and prescribing pharmacists were mentioned as groups for whom the intervention might be less applicable due to their specific needs or roles.

staff groups… I mean most would probably benefit it’s a bit more streamlined particularly for things you don’t prescribe frequently. Participant 22

I think it would be useful everywhere to be honest… like having tools like this are always helpful. Participant 15

## Discussion

This study investigated the efficacy of a patient-specific, indication-based prescribing tool in reducing prescribing errors, improving prescribing efficiency and alleviating user workload compared with standard practice. The results show a substantial reduction in prescribing errors and median and upper TTP quartiles when using the intervention. The hierarchical task analysis identified workflow vulnerabilities related to errors. The intervention mitigated many error types seen in the control arm by streamlining access to patient-specific information, automating dose calculations and providing clear dose recommendations. However, challenges remained in broader prescribing workflows, such as correctly launching the patient in the electronic health record system and transcribing the correct dose for specific routes of administration. User feedback and NASA-TLX scores confirmed the intervention’s positive impact on user experience and workload.

This study’s findings align with growing evidence that indication-based prescribing systems can reduce prescribing errors and improve efficiency.^6 9 10 26^ Our results closely match those from Garabedian *et al*^10^ particularly in terms of error rates and task time when using these systems versus standard practices. Despite differences between US and UK healthcare systems, these combined findings support the adoption of indication-based prescribing systems across various healthcare settings.

As for the earlier US study,^10^ this study provides further evidence that demonstrates the potential of indication-based prescribing tools in a simulation environment. The positive feedback from participants suggests a higher likelihood of acceptance in clinical settings, as predicted by technology acceptance models.^27^,^29^ Ongoing user feedback will be essential for refining future prototypes and ensuring successful implementation across diverse settings and patient groups.

A key aspect of our user-testing process was identifying system and workflow vulnerabilities, which should lead to further error reduction. Similar studies on medication-related prescribing and administration guidance have shown the effectiveness of this approach.^30 31^ However, small or large changes to individual interventions alone may not ensure widespread adoption of these tools. According to Schiff *et al*,^8^ larger scale, ‘radical change’ and clinician buy-in are necessary. We propose that buy-in at all levels—from patients to prescribers, senior management, and procurement teams—is crucial for implementing and scaling these systems. Given the increasing demands on healthcare services,^32^ engaging with cautious senior management will require robust evidence to support new interventions.

### Strengths and limitations

The study’s strengths lie in its broad medication scenario selection, diverse participant pool, objective outcome measures, comprehensive analysis methods and robust statistical analysis. The use of a concurrent triangulation design method allowed for the collection of data and analysis using a combination of methods over a shorter period of time compared with a sequential approach.^11^ This is the first evaluation of its type in England of a patient-specific, indication-based prescribing tool and aligns with similar work from the USA.^10 26^

However, the study has several limitations. The simulation setting could not fully replicate the complexities of real-world clinical practice, and the relatively small sample size of 24 participants limits the statistical power of the findings. Furthermore, conducting the research within a single organisation and comparing it to a single eP system may limit its generalisability. In addition, nurse prescribers invited to participate were unable to do so, due to clinical workload. Participants' responses may have been influenced by specific components of the intervention, such as dose calculation or indication-based prescribing. As a result, comparing the intervention to other systems with similar features could affect participants' overall perceptions. Additionally, the study was conducted before the intervention was fully integrated with the EHR, requiring further testing post integration to ensure the system is fast enough for clinical use and performs as expected. Last, further user testing of the indication search and ‘indication-first prescribing’ workflow is needed. Future research should address these limitations.

### Recommendations for research and practice

Based on the study findings, we make several recommendations in relation to this intervention. There is a need for further refinement of the interface and deeper integration with the local electronic health record. This could help mitigate risk of wrong-patient errors. Additionally, incorporating the ability to ‘push’ the final dose recommendation directly into the medication order form could reduce transcription errors. Real-world evaluations will be crucial to assess the intervention’s impact on clinical outcomes and prescriber workflows. These evaluations should involve diverse healthcare settings and patient populations to ensure generalisability and scalability. More broadly, CDSS tools should integrate with local prescribing guidelines to ensure alignment with institutional protocols and to help clinicians make informed decisions based on local best practices. Development of comprehensive training is essential to ensure that clinicians are proficient at using new interventions, training should include system navigation, interpretation of recommendations and how to integrate the system into existing workflows to maximise user adoption and minimise errors.

## Conclusion

This study demonstrates the potential of an indication-based, patient-specific prescribing tool, to reduce error, improve efficiency and reduce user workload in healthcare settings. The findings underscore the importance of integrating human factors and usability engineering principles into the development of CDSS to optimise user experience and effectiveness. Indication-based, patient-specific prescribing tools represent a promising advancement in medication safety technology, with implications for enhancing patient care and healthcare system efficiency.

## Supplementary material

10.1136/bmjqs-2024-017733 online supplemental file 1

## Data Availability

Data are available on reasonable request.
