# Supplementary material for: Comparing safety, performance and user perceptions of a patient-specific indication-based prescribing tool with current practice: a mixed methods randomised user testing study
Source: BMJ Qual Saf. 2024 Nov 21;34(11):e017733. doi: 10.1136/bmjqs-2024-017733 (PMC12573381; doi:10.1136/bmjqs-2024-017733)

Online Supplementary Information

Appendix 1 – *Concurrent triangulation methods applied to this study*

Appendix 2 - *User interface of intervention tool Touchdose - indication selection page for omeprazole*

Appendix 3 - *User interface of intervention tool Touchdose - selected dose recommendation page for omeprazole. Patient-specific, indication-based dose recommendation presented to user.*

Appendix 4 - *Block randomisation groups*

Appendix 5- *Simulated scenarios for Group 1 (order of medication set and intervention arm determined by group)*

Appendix 6 - *Semi-structured interview topic guide*

Appendix 7 - *Error scoring example - Prescribing scenario, anticipated answer and potential errors as presented to error scoring panel for discussion.*

Appendix 8 - *Univariate and multivariate lower (25th) quantile regression outputs for models examining the relationship between the variable of interest (Arm), the covariates (Period and Set) and time to prescribe*

Appendix 9 - *Univariate and multivariate median quantile regression outputs for models examining the relationship between the variable of interest (Arm), the covariates (Period and Set) and time to prescribe*

Appendix 10 - *Univariate and multivariate upper (75th) percentile regression outputs for models examining the relationship between the variable of interest (Arm), the covariates (Period and Set) and time to prescribe*

Appendix 11 - *Time to prescribe box and whisker plot comparison by arm*

Appendix 12 - *Hierarchical task analysis for the control arm erroneous medication orders.*

Appendix 13 - *Hierarchical task analysis for the Touchdose (intervention) arm erroneous medication orders*

Appendix 14 - *NASA-Task load index (TLX) results by arm and TLX domain*

Appendix 15 - *Box and whisker plots of NASA Task load index (TLX) overall score across all domains, for control and intervention (Touchdose) arms*

Appendix 16 - *Box and whisker plots of NASA Task load index (TLX) scores, by each domain, for control and intervention (Touchdose) arms*

Appendix 1 – Concurrent triangulation methods applied to this study

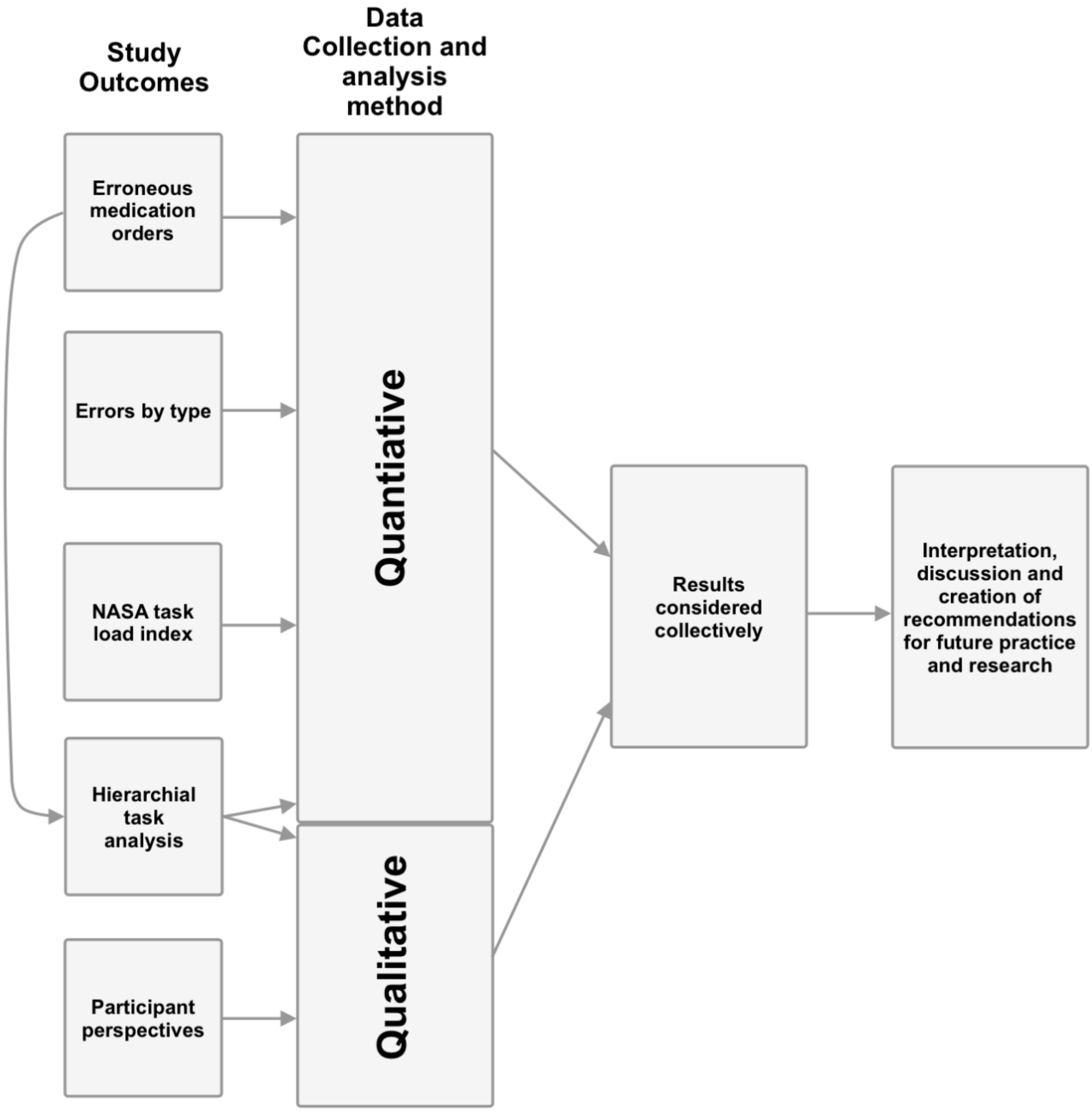

Appendix 2 User interface of intervention tool Touchdose - indication selection page for omeprazole

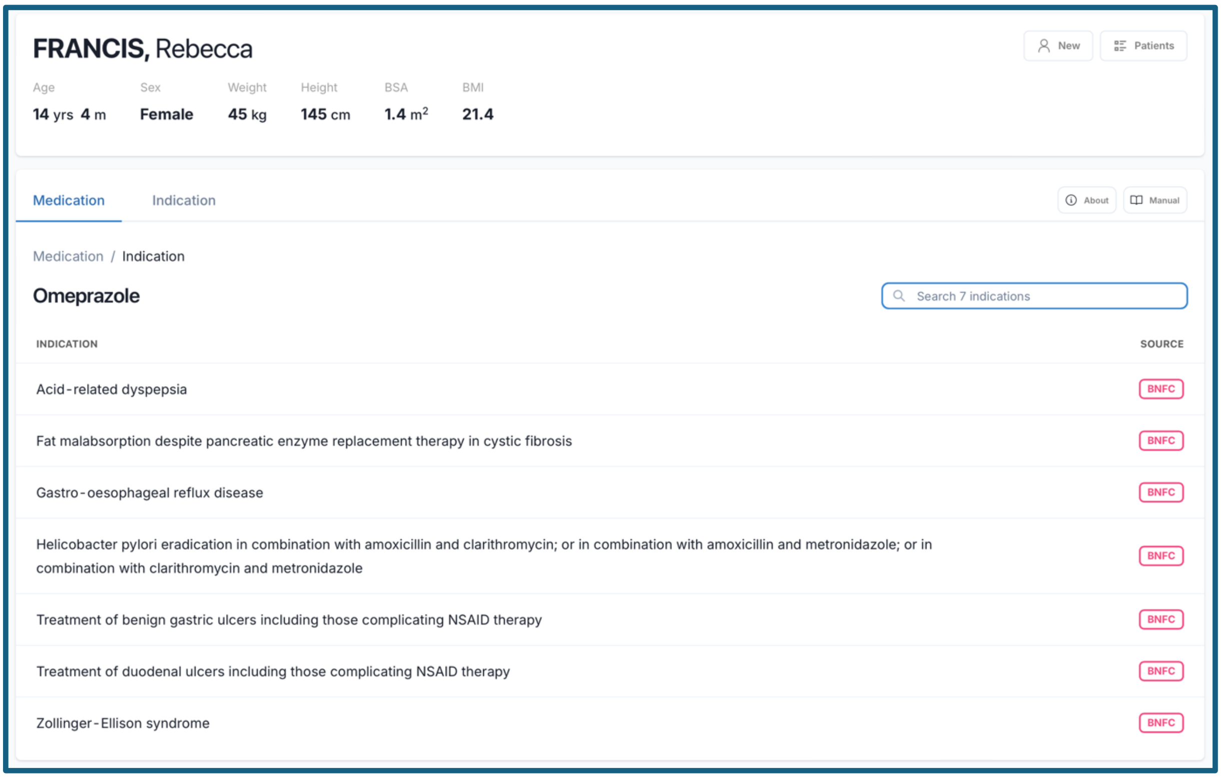

Appendix 3 User interface of intervention tool Touchdose - selected dose recommendation page for omeprazole. Patient-specific, indication-based dose recommendation presented to user.

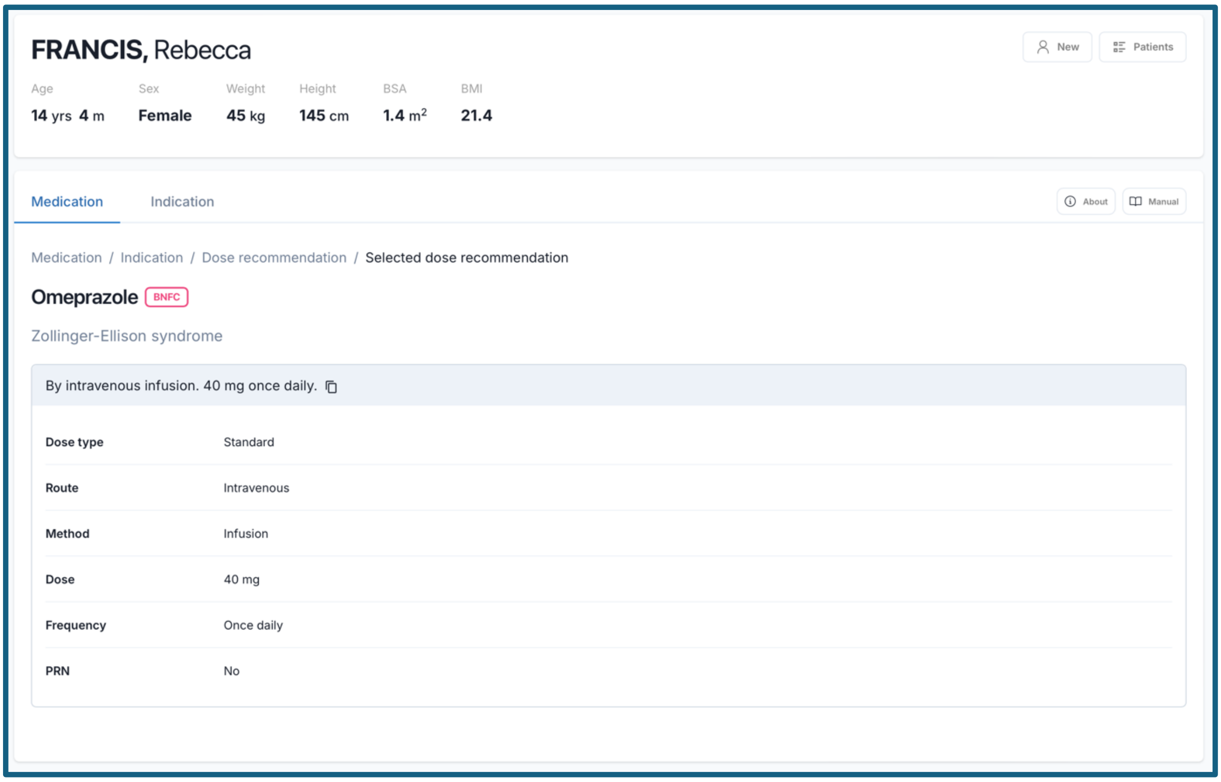

Appendix 4 Block randomisation groups

	Group 1 (n = 6)	Group 2 (n = 6)	Group 3 (n = 6)	Group 4 (n = 6)
Period 1	Set 1<br>Control	Set 2<br>Intervention	Set 1<br>Intervention	Set 2<br>Control
Period 2	Set 2<br>Intervention	Set 1<br>Control	Set 2<br>Control	Set 1<br>Intervention

n = number of participants randomised to each group that participated in the study

Appendix 5 Simulated scenarios for Group 1 (order of medication set and intervention arm determined by group)

Group 1									
		**Name**	**Patient information**	**Date of Birth**	**Medical Records Number**	**Medication**	**Indication**	**Route required**
**Practice**	**Intervention**	Susan Burnett	Female, 60 years, 58kg, 140cm	07/07/1962	31437555	Lorazepam	Anxiety	Oral
		John Aspen	Male, 20 years, 98kg, 170cm	07/07/2002	31493904	Tobramycin	Sepsis, severe infection	IV
**Set 1**	**Contol**	Bob Wills	Male, 75 years, 80kg, 170cm	07/07/1947	31437471	Diltiazem (Dilzem brand) SR	Angina – starting dose	Oral
		Nicholas Apple	Male, 55 years, 98kg, 175cm	07/07/1967	31437542	Mercaptopurine	Chronic Myeloid leukaemia, initial dose, unable to take tablets	Oral, *liquid
		Rebecca Francis	Female, 13 years, 45kg, 145cm	07/07/2009	31437497	Omeprazole	Zollinger-Ellison syndrome	IV
		Steve Knight	Male, 20 years, 98kg, 170cm	07/07/2002	31437474	Aciclovir	Treatment of Herpes zoster infection	IV
		Elliot Holmes	Male, 18 years, 67kg, 185cm	07/07/2004	31437476	Fosfomycin	Osteomyelitis when first-line treatments are inappropriate or ineffective	IV
**Please complete Questionnaire before continuing**								
**Set 2**	**Intervention**	Jennifer Collins	Female, 60 years, 58kg, 155cm	07/07/1962	31437530	Levofloxacin	Chronic pulmonary infections due to Pseudomonas aeruginosa in cyctic fibrosis	Neb
		Alfred Allan	Male, 11 years, 37kg, 130cm	07/07/2011	31437494	Caspofungin	Invasive candidiasis, Loading dose	IV
		Marius Dixon	Male, 6 months, 8.5kg, 69cm	07/05/2022	31437552	Omeprazole	Zollinger-Ellison syndrome, severe reflux not resolved on Omeprazole 10mg OD	Oral
		John Smith	Male, 60 years, 117kg, 200cm	07/07/1962	31437468	Vancomycin	Cellulitis – initial loading dose	IV
		Lena Herring	Female, 11 years, 50kg, 1.54m	07/07/2011	31437553	Azithromycin	Prevention of secondary case of invasive group A streptococcal infection, in patients who are allergic to penicillin.	Oral
**Please complete Questionnaire before you leave**								

Legend: kg- kilograms IV - intravenous, Neb- nebuliser, OD- once daily

Appendix 6- Semi-structured interview topic guide

Touchdose user testing questions-

- Please tell me how you found today's user testing session.
    - Could you describe how you found using Touchdose to complete the prescribing tasks.
    - How did this compare with your current prescribing practice?
- What effect do you think a system such as Touchdose could have on your prescribing practice?
    - If positive, in what way?
    - If negative, in what way?
    - Is there anything that you think would benefit from changing/improving?

Indication-based prescribing workflow questions –

- Did you utilise the option to search by indication as opposed to by medication first?
- How does this align with your current prescribing workflow, both your mental workflow and decision making as well as the physical act of prescribing, for example entering the information onto the prescription?
- Would you consider an indication-*first* prescribing workflow to be better/worse/the same as current prescribing workflows. For example, searching for an indication and then selecting a medication, rather than searching for the medication.

Probes –

- Can you expand on that
- Do you have any examples –
    - where this would have been useful...?
    - where this may have hindered...?
    - specific patient groups
    - specific medication types – antimicrobial stewardship

Appendix 7 Error scoring example - Prescribing scenario, anticipated answer and potential errors as presented to error scoring panel for discussion.

Name	Patient info	DOB	Medication	Indication	Route required
Rebecca Francis	Female, 13 years, 45kg, 145cm	07/07/2009	Omeprazole	Zollinger-Ellison syndrome	IV

Anticipated answer – Omeprazole, 40mg, OD, IV, infusion

27	60mg, OD, IV, infusion
28	20mg, OD, oral, administer most appropriate formulation
29	20mg, once only, oral, gastro-resistant capsule
30	20mg, OD, oral, gastro-resistant capsule
31	20mg, OD, IV, infusion

Appendix 8 Univariate and multivariate lower (25th) quantile regression outputs for models examining the relationship between the variable of interest (Arm), the covariates (Period and Set) and time to prescribe

Time to prescribe *25th Quartile regression*	Univariate Model			Multivariate Model		
	Coef (s)	95% confidence interval	p value	Coef (s)	95% confidence interval	p value
Arm						
**Control**						
Intervention	-17	-35.07 – 1.07	0.07	-14	-32.40 – 4.40	0.14
Period						
Period 1						
**Period 2**	14	-3.03 – 31.03	0.11	11	-7.4 – 29.40	0.24
Set						
**Set 1**	-17	-35.59 – 1.58	0.07	-11	-29.33 – 7.33	0.24
Set 2						

Coef – coefficient, (s)- seconds. **Bold** indicates reference category.

Appendix 9 Univariate and multivariate median quantile regression outputs for models examining the relationship between the variable of interest (Arm), the covariates (Period and Set) and time to prescribe

Time to prescribe *Median quartile regression*	Univariate Model			Multivariate Model		
	Coef (s)	95% confidence interval	p value	Coef (s)	95% confidence interval	p value
Arm						
**Control**						
Intervention	-35	-62.42 – -7.54	0.01	-36	-63.87 – -8.12	<0.01
Period						
Period 1						
**Period 2**	19	-5.77 – 43.77	0.13	15	-12.88 – 42.88	0.29
Set						
**Set 1**	-17	-43.21 – 9.2	0.20	-21	-48.76 – 6.76	0.14
Set 2						
Coef – coefficient, (s)- seconds. **Bold** indicates reference category.						

Appendix 10 Univariate and multivariate upper (75th) percentile regression outputs for models examining the relationship between the variable of interest (Arm), the covariates (Period and Set) and time to prescribe

Time to prescribe 75th Quartile regression	Univariate Model			Multivariate Model		
	Coef	95% confidence interval	p value	Coef	95% confidence interval	p value
Arm						
**Control**						
Intervention	-56	-103.75 – -8.25	0.02	-80	-127.17 – -32.82	<0.01
Period						
Period 1	36	-7.88 – 79.88	0.11	43	-4.17 – 90.17	0.07
**Period 2**						
Set						
**Set 1**	-17	-63.20 – 29.20	0.47	-18	-64.97 – 28.98	0.45
Set 2						
Coef – coefficient, (s)- seconds. **Bold** indicates reference category.						

Appendix 11 Time to prescribe box and whisker plot comparison by arm

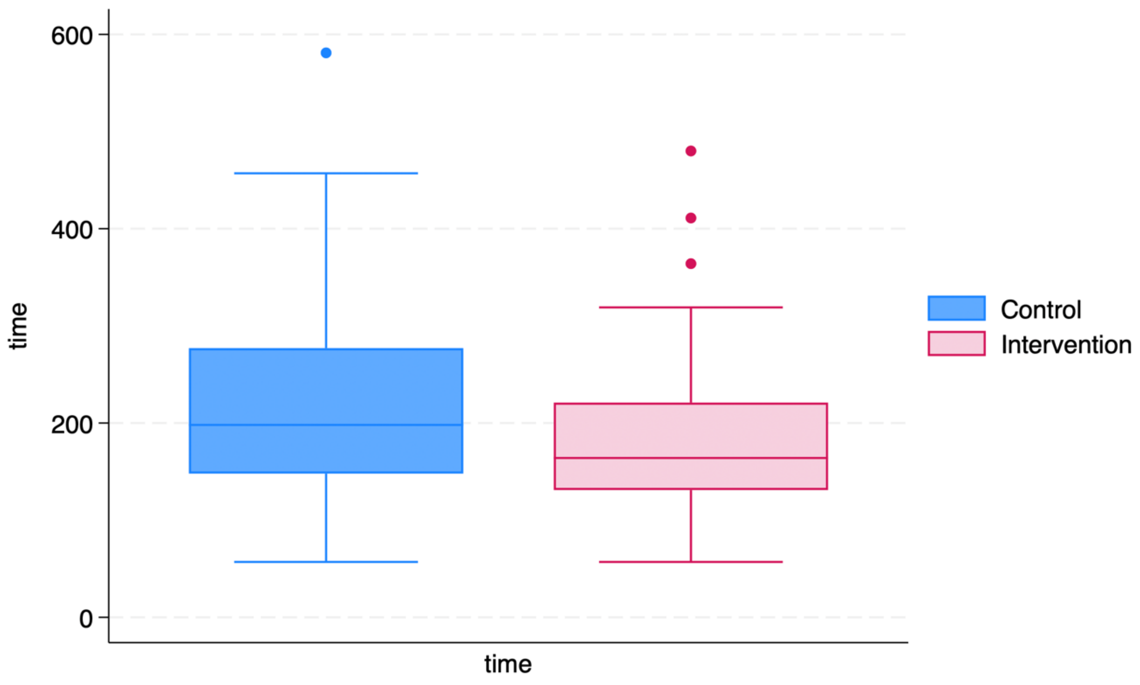

Appendix 12 Hierarchical task analysis for the control arm erroneous medication orders. BNF: British National Formulary. IBW: ideal body weight. BSA = body surface area.

**Medication**	**Note**	**Step(s) associated as contributory factor to error (number relates to workflow flow chart)**	**Number of occurrences across Control arm**
**Aciclovir**	Failure to identify dose requires calculation using IBW as patient obese	3.3/4.4	4
**Aciclovir**	Failure to identify patient specific route requirement on scenario, therefore dose, route, frequency incorrect	0/3.2	1
**Aciclovir**	Failure to calculate dose based on IBW, inappropriate rounding of dose. (Correctly identified that IBW dose was required)	4.3/5.3	1
**Caspofungin**	Failure to identify and apply max dose to BSA calculated dose	3.3/4.4	4
**Caspofungin**	Failure to identify and apply loading dose (lower maintenance dose prescribed)	0 / 3.2	1
**Caspofungin**	Failure to calculate correct dose using BSA	4.4	1
**Caspofungin**	Failure to identify and apply max dose to BSA calculated dose	3.3/4.4	1
**Diltiazem**	Dose for adult patient given rather than elderly patient (patient aged 75years)	3.2	3
**Diltiazem**	Failure to identify brand specific dosing recommendation, leads to dose, form, brand error	3.2, 5.4/5.8/5.9	1
**Diltiazem**	Adult dose recommendation transcribed from BNF, using partially completed order sentence for a specific Brand. Therefore dose, brand and frequency incorrect.	3.2/5.3	1
**Diltiazem**	Failure to identify brand specific dosing recommendation, leads to dose and brand error	0 / 3.2	1

**Diltiazem**	Failure to identify brand specific dosing recommendation and elderly dose recommendation, therefore dose, frequency and brand error	3.2/ 5.9	1
**Fosfomycin**	Failure to identify and apply max dose limit	3.3/4.4	1
**Levofloxacin**	incorrect route, BNF stated inhalation of nebulised solution. order states route inhalation, form solution.	5.6	1
**Mercaptopurine**	Form error, prescribes tablets instead of oral suspension. Failure to identify patient specific needs from scenario, (it is also the incorrect dose for by tablet)	0/3.2	1
**Mercaptopurine**	Failure to identify patient specific form requirement on scenario, therefore dose and form incorrect.	0/3.2	1
**Mercaptopurine**	Selection of a partially completed order sentence with tablet as pre-specified form, failure to edit form to patient specific route requirement.	5.8	1
**Mercaptopurine**	Failure to calculate dose per meters squared and to input dose correctly ("25mg/m" entered as dose)	4.3/5.4	1
**Omeprazole A**	Failure to identify patient specific route requirement on scenario, therefore dose, route and form incorrect.	0/3.2	2
**Omeprazole A**	Using BNF for paediatric patient, prescribes adult dose	3.1/3.2	1
**Omeprazole A**	Failure to identify and apply route specific dose recommendation	3.2	1
**Omeprazole B**	Failure to identify and apply max dose limit	3.3/4.4	2
**Omeprazole B**	Failure to identify patient specific information regarding current dose on scenario, therefore dose error as dose prescribed was less than patients current dose and failure to 'increase if necessary to 20mg'	0/3.2	1
**Vancomycin**	Failure to calculate correct per kilogram dose	4.3	1

Appendix 13 Hierarchical task analysis for the Touchdose (intervention) arm erroneous medication orders

Medication scenario	Contributory factor to error	Step(s) associated as contributory factor to error (number relates to workflow flow chart)	Number of occurrences across the intervention arm
**Omeprazole A**	Failure to identify patient specific route requirement on scenario, therefore dose, route and form incorrect.	0	3
**Omeprazole B**	Failure to identify patient specific information regarding current dose on scenario, therefore dose error as dose prescribed was less than patients current dose and failure to 'increase if necessary to 20mg'	0 / (5.3)	2
**Azithromycin**	Failure to launch required patient in Cerner (participant prescribed medication for the previous patient).	1.1	1
**Mercaptopurine**	Failure to input a single dose (participant prescribed dose as a range)	6.5	1
**Mercaptopurine**	Incorrect dose for route prescribed	5.3 / (6.5)	1

Appendix 14 NASA-Task load index (TLX) results by arm and TLX domain

Group		Overall task load	Mental demand	Physical demand	Temporal demand	Performance	Effort	Frustration
Control	Mean	57.21	12.9	4.66	8.87	7.95	12.08	10.7
Intervention	Mean	41.45	9.5	3.25	6.66	7.62	8.25	6.17
Mann-Witney	P value	<0.01	0.03	<0.30	0.06	<0.44	<0.01	<0.01

Appendix 15 Box and whisker plots of NASA Task load index (TLX) overall score across all domains, for control and intervention (Touchdose) arms

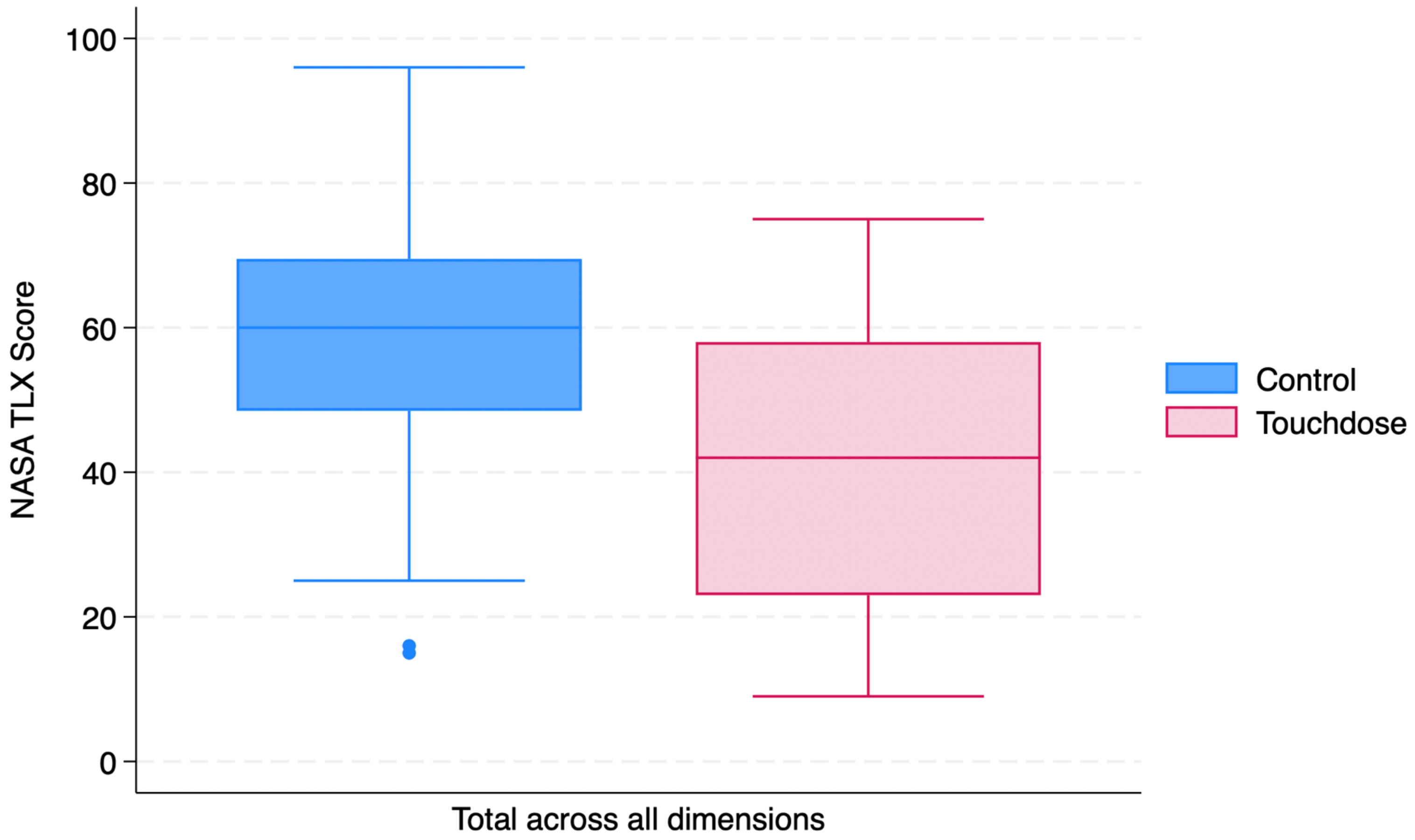

Appendix 16 Box and whisker plots of NASA Task load index (TLX) scores, by each domain, for control and intervention (Touchdose) arms

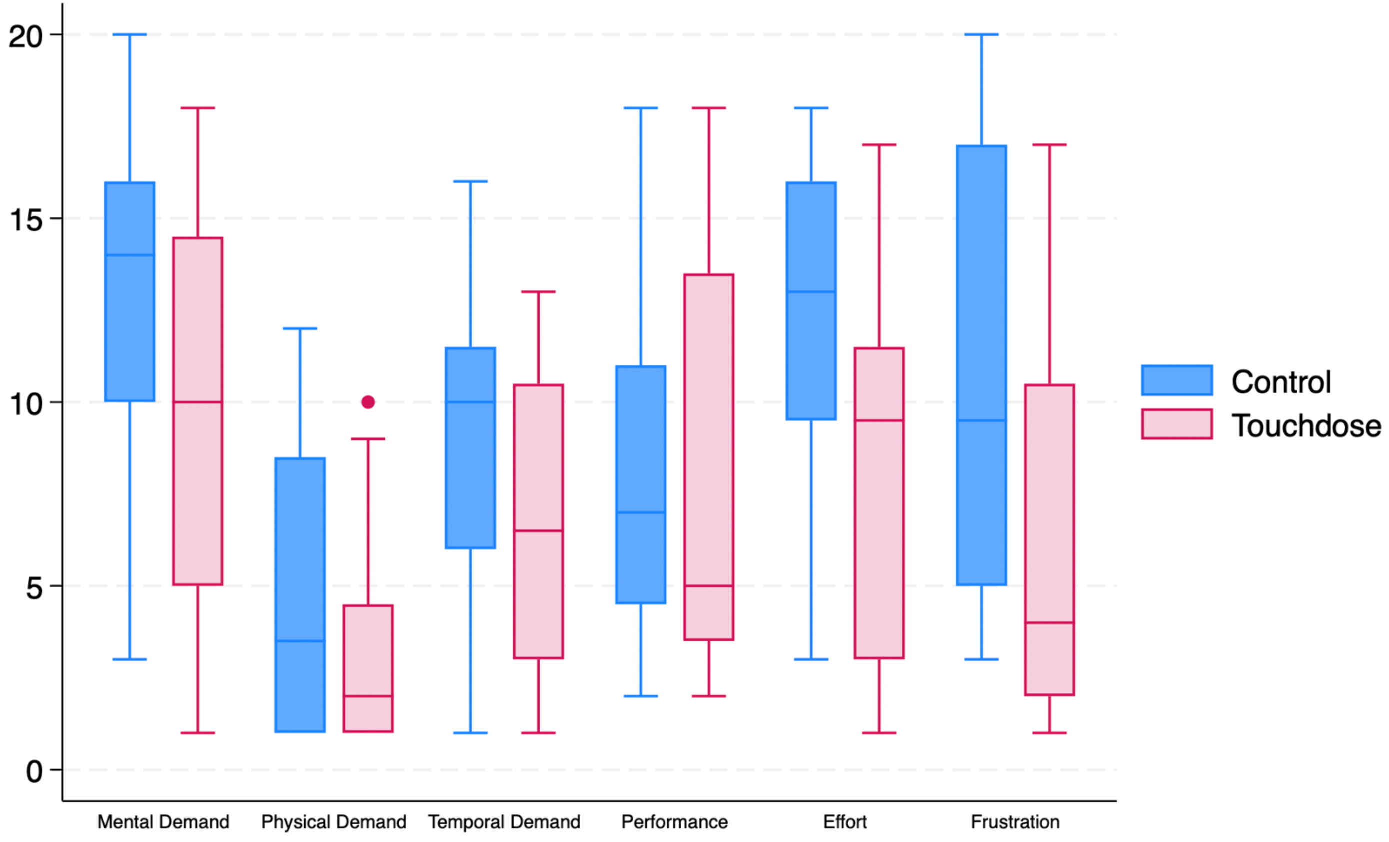

Supplement: online supplemental file 1 [file bmjqs-34-11-s001.pdf]
